# Contribution to unravel variability in bowhead whale songs and better understand its ecological significance

**DOI:** 10.1038/s41598-020-80220-5

**Published:** 2021-01-08

**Authors:** F. Erbs, M. van der Schaar, J. Weissenberger, S. Zaugg, M. André

**Affiliations:** 1 Laboratory of Applied Bioacoustics, Technical University of Catalonia, BarcelonaTech (UPC), Barcelona, Spain; 2 FT SST ERO, Equinor ASA, Oslo, Norway

**Keywords:** Animal behaviour, Behavioural ecology

## Abstract

Since the first studies on bowhead whale singing behaviour, song variations have been consistently reported. However, there has been little discussion regarding variability in bowhead whale singing display and its ecological significance. Unlike the better studied humpback whales, bowhead whales do not appear to share songs at population level, but several studies have reported song sharing within clusters of animals. Over the winter season 2013–2014, in an unstudied wintering ground off Northeast Greenland, 13 song groups sharing similar hierarchical structure and units were identified. Unit types were assessed through multidimensional maps, showing well separated clusters corresponding to manually labelled units, and revealing the presence of unit subtypes. Units presented contrasting levels of variability over their acoustic parameters, suggesting that bowhead whales keep consistency in some units while using a continuum in values of frequency, duration and modulation parameters for other unit types. Those findings emphasise the need to account for variability in song analysis to better understand the behavioural ecology of this endangered species. Additionally, shifting from song toward units or phrase-based analysis, as it has been suggested for humpback whales, offers the opportunity to identify and track similarities in songs over temporal and geographical scales relevant to population monitoring.

## Introduction

Bowhead whales (*Balaena mysticetus*) are highly vocal animals and during the coldest and darkest Arctic winter months they exhibit an elaborated acoustic display that rivals the complexity of the better known humpback whale songs^1^. Although the sex of the singers and the song function in bowhead whales are still unknown, it has been hypothesised that singing behaviour plays a major role in mediating sexual interactions^1–5^ in a way similar to humpback whales and songbirds^6,7^.

Bowhead whale songs have been described following the same hierarchical framework established to study humpback whales, with sequences of repeated units composing phrases, phrases composing themes, and themes composing a song^1^. Contrarily to humpback whales that share song within the same population, bowhead whales appear to share songs within a limited cluster of animals^5,8^. In the first review of the earliest acoustic researches on bowhead whales Würsig and Clark^1^ described a ‘great deal of intra and inter individual song variation’ and the following studies have consistently reported song variability. The study of the variation in behavioural traits such as the acoustic repertoire can provide insights on how sexual selection acts, at individual, group, population or species levels, and shape acoustic phenotypes (i.e. songs). For example, song variations in humpback whales have been used to investigate the function of song components in the context of inter- and intrasexual selection^9,10^, to examine population connectivity or group dynamics (e.g.^11–13^), and to investigate mechanisms underpinning gradual song evolution and cultural revolution (e.g.^14–16^).

In bowhead whales, variability is similarly present at different levels of the hierarchical song structure. At the unit level, the unit contour shape can change through splitting, missing components, frequency shifts, or modification in the number of inflection and modulation points^2,3,17^. Unfortunately, there are currently no clear criteria establishing the acceptable level of variability within a unit type and the classification procedure remains highly subjective. Manual or automated categorisation into unit types can be furthermore complicated by the graded nature of the bowhead whale repertoire. For example, Johnson et al.^8^ described ‘subnotes’ (subtypes of units) along a graded unit sequence (similar to gradual changes in units described in humpback whale shifting themes^18,19^), where both unit duration and frequency parameters evolved along the song. The authors eventually assigned the last units of the sequence to a different type, based on “trend of frequency”, a criterion that has not been reported in other song studies. At the song level, one of the most variable components appears to be the number of repetition of units per phrase. All studies describing song acoustic and structural parameters report on a high level of variation, with repetition rates ranging from one to tens of times, and some units being sometimes entirely omitted or rarely present in the song^3,8^. Directly linked to unit repetition, variability across renditions of the same song have been previously reported including variability in song duration along a song bout^2^ and the occurrence of ‘primitive versions’ during the initial development of a song as well as song variants based on unit or phrase repetition, order or absence^3,8^. Similar variants have been recently identified as “song fragments” in humpback whales songs^20^. Collectively, the bowhead whale acoustic studies indicate that variability at multiples levels is an important part of the bowhead whale singing behaviour. Although this variability has been consistently reported either as a qualitative descriptor of songs^1,21^ or with a statistical measure of dispersion in previous publications^2,3,8,17^, the study of variability itself has never been the central subject of bowhead whale acoustic research.

Research challenges related to the bowhead whale Arctic distribution have constrained the advances of acoustic studies and although the first reports of bowhead whale songs date back to the early 1980s there have only been a handful of publications since then describing their songs. Many of those studies have focused on song diversity using the number of different songs as a single metric^2,3,5,8,21,22^. At first glance, counting songs might seem straightforward, but defining at which point a song is different enough to be considered new represents a delicate problem when the signals to be categorised display high variability. Furthermore, integrating variability in the definition of what is new or different is essential to delve into the potential functions of a song and advances our understanding of singing behaviour in bowhead whales.

Here we used a one-year dataset that sampled bowhead whales from the critically endangered Spitsbergen population in a previously undescribed overwintering area in Northeast Greenland coastal waters to examine song variability. We selected songs shared by multiple individuals as a basis to focus on inter and intra individual aspects of variability. The shared songs, displaying comparable song structure and unit acoustic characteristics, were used to identify patterns of variations using semi-automated methods. We explored the potential ecological and behavioural significance of this song variability within the bowhead whale mating context.

## Methods

### Study site and data collection

**Figure 1:**
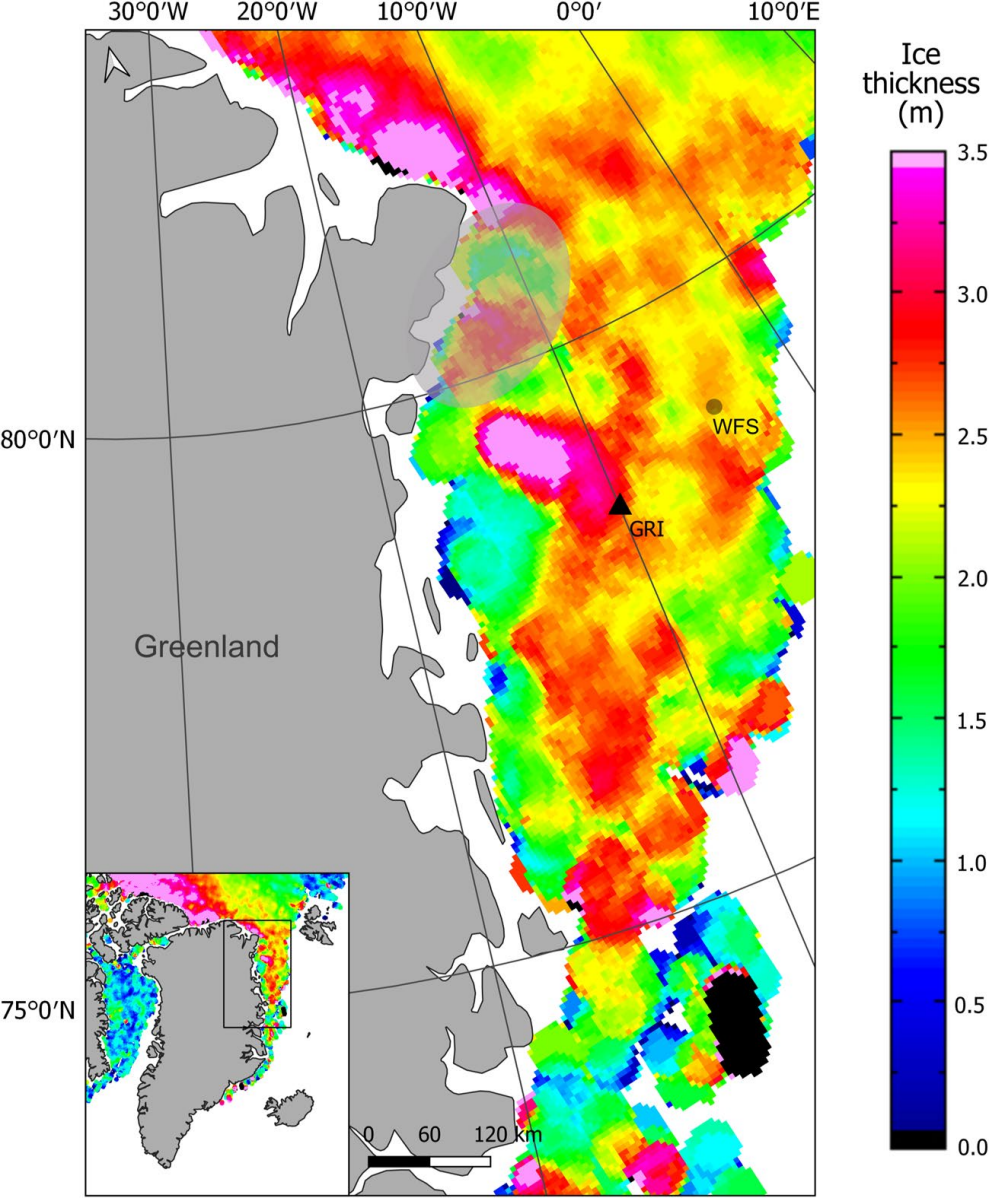
Map of the North East Greenland coast showing the Greenland I (GRI) recording location. Colours as shown in legend represent sea ice thickness values for December 2013 (data from CryoSat-2^68^). The grey dot is the location of the Western Fram Strait recording location from previous studies mentioned in the text^22,33^. The grey ellipse represents the position and extent of the Northeast Water polynya. (Map created using QGIS v.3.8.3, https://www.qgis.org/).

Acoustic data were collected in coastal waters of Northeast Greenland (78°30′N and 10°0′W) on the continental shelf about 170 km offshore, close to the Northeast Water (NEW) polynya (Fig. 1). The NEW is an area of major interest as a summering ground for the Spitsbergen bowhead whale population^23^.

The recording equipment was deployed during the Oden Artic Technology Research Cruise 2013 on the 23th of August 2013 and retrieved on the 17th of September 2014. The hydrophone, an AGUAtech Low-Power Scientific Measurement Hydrophone (sensitivity -160 dB re 1 V/μPa), was directly connected to the acoustic recorder, an RTSys EA SDA14 (RTsys, Caudan, France). The system was suspended a few tens of metres above the sea floor using a subsurface float, in a water depth of 227 m. The recording was set on a duty cycle of 2 min on and 30 min off, at a sampling frequency of 39,062 Hz in 24 bits. The data was hosted in the LIDO (http://listentothedeep.com) framework and software package^24^.

### Data annotation and measurements

**Figure 2:**
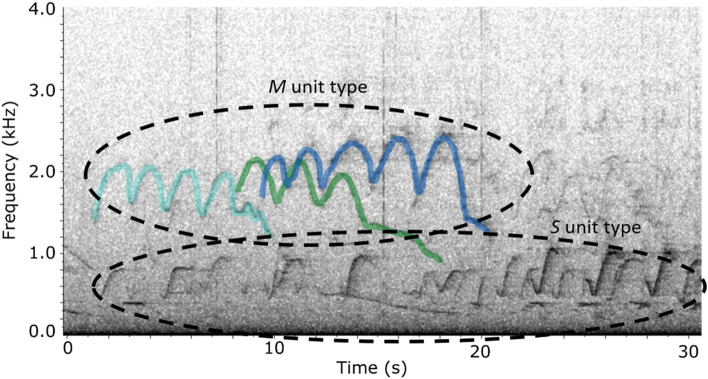
Spectrogram screenshot of an example of 3 bowhead whales sharing similar songs, recorded on the 10th of December 2013. The 3 Units M are shown in colour to highlight their overlap in time.

The dataset was first subsampled to 25% (one out of four 2-min files) and then explored to detect the occurrence of shared songs. Files were aurally and visually analysed by an expert in Adobe Audition (Adobe Systems Inc., San José, CA, USA). Each 2-min file was displayed as a spectrogram (2048 point FFT, 75% overlap, Hann window for a frequency resolution of 19 Hz and a time resolution of 52 ms). The songs shared by multiple animals were identified based on two criteria: the songs overlapped in time and contained similar unit types temporally arranged in similar phrases. Figure 2 shows an example of three units with similar acoustic characteristics (frequency contour shape, medium frequency) overlapping in time with each other (i.e. a second unit starts while a first unit has not ended yet), and associated with lower frequency units also sharing between them similar acoustic characteristics. This indicates that three individuals are sharing similar songs.

Two unit types were consistently present in most of the detected shared songs: a long whistle-like unit displaying multiple inflections (thereafter referred to as *M* unit type) and a short whistle-like unit usually containing one inflection (*S* unit type). *M* and *S* units were temporally associated in a phrase, that was usually repeated multiple times. Subsequently, the analysis was focused only on the songs containing *M* and *S* units. All the song occurrences containing the *M* and *S* units were identified from the subsampled dataset. Songs were manually classified into song groups. Song groups were defined as songs presenting a high level of similarity on the following aspects of the song characteristics and structure: overall similarity of the frequency contours of the units *M* and *S*, presence of others unit types, order of units, and biphonation characteristics if present (overview given in Table 1). The units were named after the overall shape of the frequency contours, and units belonging to a particular song group were designed by adding the song group to their name (e.g. *MSG11* refer to the units *M* from the song group SG11).

**Table 1:** Presence of unit types per song group. The order of the units in the table reflects the temporal arrangement of units composing the song groups. Presence marked with an x, absence with an empty cell.

Song group	Nb. of distinct units	*rumble*	*M*	*M* *inter*	*S*	*Vhigh*	*sLF* *down*	*sLF* *const*	*sLF* *const2*	*sup*
SG1	2		X		X					
SG5	2		X		X					
SG9	3		X		X	X				
SG12	3		X	X	X					
SG2	4	X	X		X	X				
SG4	4		X		X			X		X
SG11	4	X	X		X	X				
SG7	5		X		X	X	X		X	
SG3	5		X	X	X		X		X	
SG13	6	X	X		X	X	X		X	
SG6	6		X	X	X	X	X	X		
SG8	6	X	X		X	X	X	X		
SG10	6	X	X		X	X	X	X		

Manual analysis was then conducted on all files from the same days where the songs were initially identified to obtain more occurrences of these song groups. All occurrences, except the ones with a poor signal-to-noise ratio, were selected for further analysis. The minimum number of occurrences for a song group was three, the maximum was eight.

### Measurement of unit’s parameters

In order to reduce subjectivity inherent to human observer categorisation, we investigated unit classification and variability using a semi-automated procedure based on unit frequency, modulation and duration measurements. For each song group, all units occurring in a song were assessed for inclusion in further analysis. A unit was selected if it was complete without overlap with other signals. Spectrogram measurements of units comprising the songs were performed in Raven (version 1.5.0, Bioacoustics Research Program^25^). Some of the measured units displayed biphonation, a nonlinear phenomenon consisting of the simultaneous production of two independent frequencies^26^. Due to degraded signal to noise ratio in the lower frequencies, biphonated components were not measured in Raven, but an estimation of their frequency bandwidth and duration were noted to allow comparison and define biphonation types. The unit measurements consisted of frequency, modulation and duration parameters that are commonly used in literature describing or classifying animal vocalisations. These parameters included minimum frequency (1), maximum frequency (2), frequency bandwidth (3), frequency bandwidth 90% (4), and duration (5). Unit contours were extracted using the Frequency Contour Measurements tool in Raven. The extracted contours were imported in Matlab v.R2017a (MathWorks, Inc., Natick, MA, USA) and a custom-made smoothing function (moving average) was applied to remove noise. Additional parameters (frequency start (6) and frequency end (7), number of inflections (8), median frequency (9)) were automatically extracted in Matlab from the smoothed contours. To describe the distribution of the parameters in each unit, the mean, standard deviation (sd) and coefficient of quartile variation (cqv) were used. The cqv was preferred over the coefficient of variation to take into account that some distributions were leptokurtic^27^.

### Data analysis

After a normalisation process (centre by median, scale by range), the nine parameters described above were used as features (Orange data mining toolbox version 3.18.0,^28^). Multidimensional scaling maps (MDS), based on pairwise similarities, were produced to examine the pattern of acoustic variation in units. MDS was used to provide an automatic way of investigating visually the grouping of units into clusters and to determine whether unit types fell into distinct subtypes without stipulating a priori categories. MDS analysis allows to view the clustering tendency of the data in a few dimensions, without forcing a certain number of clusters as is needed for many clustering algorithms. Unit clusters from the manual analysis were plotted against the MDS map to explore further the acoustic similarity/dissimilarity patterns. Kruskall stress and R-squared values were used to assess the quality of the MDS model^29,30^. Higher R-squared values indicate that the distances between points in the high dimensional feature space correlate well with the distances between the same points in the MDS projection. Similarly, a stress value inferior to 0.05 suggest that the goodness of fit of the MDS distance model can be considered as good^29^. Correlation between the selected features and the MDS dimensions was used to provide an interpretation of the MDS dimension.

In order to compare the results to a fully automated clustering method, a hierarchical clustering (average linkage, based on Euclidean distances) was performed on the dataset. To take into account the higher variability regarding the *M* units, the dataset was split into two subsets, the *M* units and all the other units (named Except-*M*). The hierarchical clustering algorithm was run on each dataset. Cluster solutions from the semi-automated (MDS map) and fully automated method (hierarchical clustering) were assessed for performance using a cluster validity measurement, the Silhouette index. This index is an indication of clustering quality and takes values between -1 and 1. The higher the silhouette value is, the better is the quality of clustering, i.e. the intra-cluster dissimilarities are small compared to the inter-cluster dissimilarities^31,32^.

## Results

**Figure 3:**
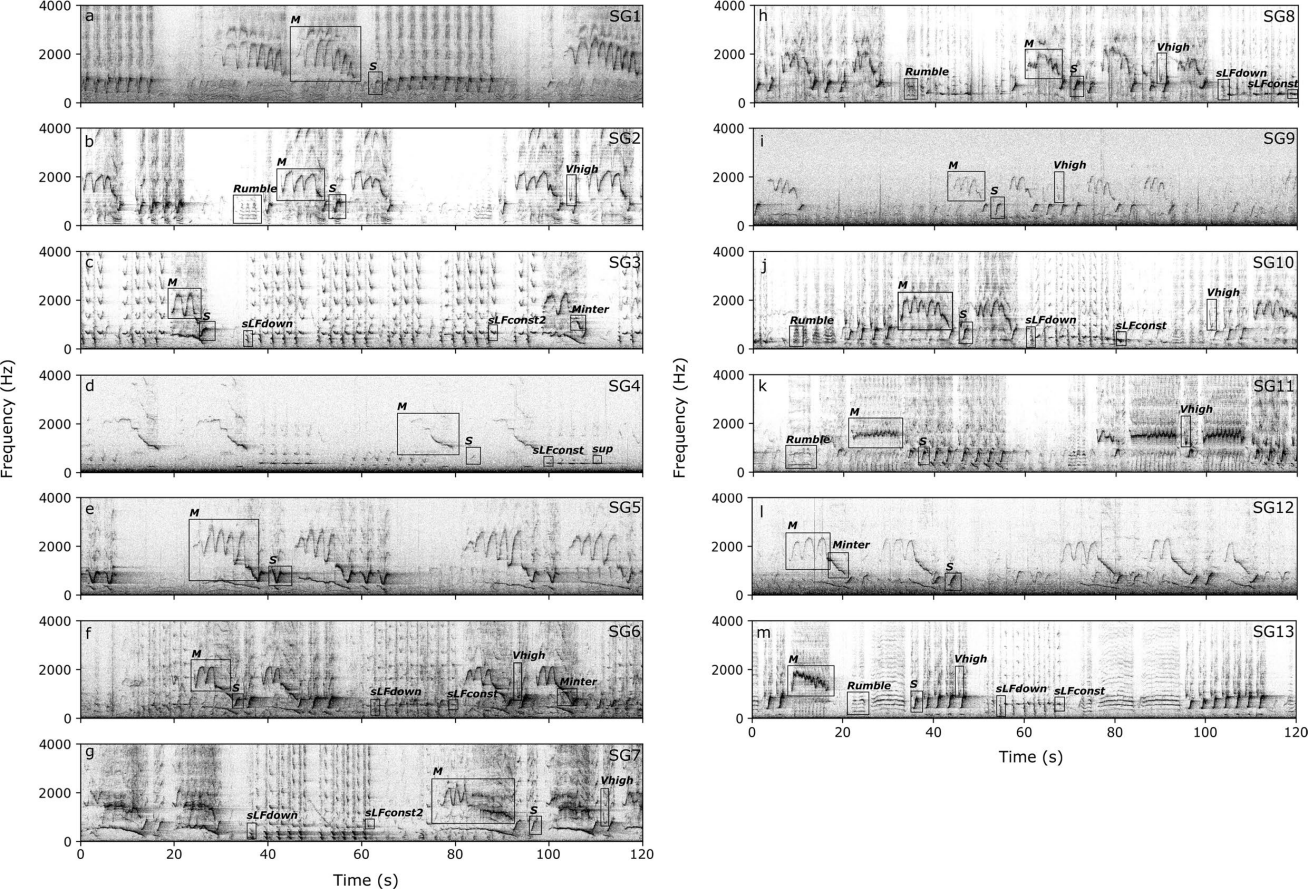
Examples of each of the 13 bowhead whale song groups recorded during December 2019 and January 2014. (**a**) SG1, (**b**) SG2, (**c**) SG3, (**d**) SG4, (**e**) SG5, (**f**) SG6, (**g**) SG7, (**h**) SG8, (**i**) SG9, (**j**) SG10, (**k**) SG11, (**l**) SG12, (**m**) SG13. Square boxes show an example of unit types present in each song. (Spectrogram parameters: 1024 pts FFT, 50% overlap, Hann window).

Bowhead whale songs were detected from the second week of October 2013 to the first week of April 2014, with almost daily detections during the peak of the season from December to February (Supplementary Fig. S1 online). These detections indicate that a probable bowhead whale mating ground described from the Fram Strait data^22,33^ could actually extend some 170 km further east towards the coast of Greenland. The manual analysis of the songs resulted in the identification of 13 song groups that comprised songs sharing similar acoustic characteristics (i.e. the presence of specific unit types *M* and *S* (see Methods and Fig. 3). The analysis revealed that up to 3 animals were singing similar songs simultaneously (Fig. 2). The song groups were detected between November and February (see Supplementary Fig. S2 online). An example of each of the song groups is shown in Fig. 3 and can be listened to on the LIDO website (link to song groups).

### Song groups description

A total of 804 units were measured and manually classified into 9 unit types using visual and audio properties of the units as well as the temporal arrangement of units in a song. An overview of the units and their main characteristics is provided in Table 2 and Supplementary Fig. S3 online, with their occurrence in song groups summarised in Table 1. Songs contained 2–6 unit types (Fig. 3 and Table 1). The overall structure of the songs consisted of two main phrases. An *M* unit and a variable number of *S* units composed the first phrase, present in all the song groups. A second phrase consisting of two alternating short and narrowband low frequency units (*sLFdown* and *sLFconst/sLFconst2*) were detected in 6 song groups, the higher frequency *sLFconst2* replacing *sLFconst* in half of them. Other units, such a s *Vhigh* and rumble-like units were present in different song groups, with the exception of *sup* occurring only in SG4.

**Table 2:** Summary of key acoustic characteristics of 9 unit types of bowhead whale songs, including mean ± standard deviation, and coefficient of quartile variation (cqv in %).

Unit type	Unit sub-type	n	Min freq (Hz) low freq	Max freq (Hz) high freq	Delta freq (Hz)	Start freq (Hz)	End freq(Hz)	Median freq (Hz)	Delta time (s)	Inflections
***M***		158	1055 ± 165 (11)	2160 ± 237 (6.1)	1105 ± 340 (16)	1724 ± 222 (25)	1142 ± 187 (11)	1672 ± 204 (10)	8.46 ± 2.4 (19)	8.8 ± 4.0 (25)
	***MSG1***	9	1077 ± 46.6 (4.4)	2069 ± 85.3 (1.7)	1532 ± 74.3 (1.7)	2002 ± 174 (4.0)	1122 ± 83.5 (3.1)	2012 ± 88.1 (3.4)	14.1 ± 1.2 (7.6)	13 ± 1.6 (7.7)
	***MSG5***	12	762.4 ± 55.3 (1.6)	2586 ± 168 (4.7)	1823 ± 176 (6.9)	1817 ± 143 (7.1)	820.2 ± 60.1 (4.1)	1894 ± 94.2 (3.9)	10.5 ± 2.2 (14)	9.1 ± 1.9 (17)
	***MSG11***	12	1271 ± 109 (4.2)	1771 ± 77.9 (1.7)	501.0 ± 114 (18)	1722 ± 87.7 (1.7)	1438 ± 141 (5.4)	1512 ± 48.5 (0.9)	9.09 ± 2.5 (6.7)	17 ± 5.7 (9.1)
	***MSG2***	17	1163 ± 55.5 (1.7)	2246 ± 62.3 (1.3)	1083 ± 72.8 (3.5)	1962 ± 98.5 (3.9)	1225 ± 53.2 (1.9)	1923 ± 58.5 (3.0)	8.84 ± 1.1 (10)	9.1 ± 1.8 (11)
	***Mo***	108	1046 ± 144 (11)	2015 ± 139 (4.9)	1060 ± 216 (13)	1654 ± 210 (8.4)	1125 ± 157 (9.0)	1598 ± 157 (6.5)	7.63 ± 1.8 (15)	7.3 ± 2.5 (29)
***S***		351	461.7 ± 76.2 (13)	872 ± 93.7 (7.3)	410.6 ± 99.7 (17)	593.0 ± 149 (17)	803.0 ± 78.0 (7.2)	728.3 ± 110 (13)	1.47 ± 0.4 (14)	1.4 ± 0.8 (33)
***Vhigh***		120	973.4 ± 103 (7.2)	1605 ± 179 (7.2)	631.6 ± 166 (19)	1461 ± 204 (11)	1155 ± 145 (9.5)	1095 ± 124 (7.2)	0.61 ± 0.2 (14)	1.0 ± 0.3 (0.0)
***rumble***		37	234.7 ± 43.7 (7.8)	319.6 ± 61.3 (7.2)	84.85 ± 26.5 (24)	278.9 ± 51.2 (6.7)	272.7 ± 52.6 (6.9)	279.5 ± 55.8 (5.1)	4.44 ± 2.4 (38)	7.3 ± 4.9 (38)
	**short R**	26	221.4 ± 43.7 (5.2)	298.0 ± 57.0 (5.1)	76.67 ± 22.1 (16)	265.9 ± 54.0 (6.9)	258.6 ± 52.9 (5.2)	257.7 ± 47.5 (5.2)	3.05 ± 0.9 (19.2)	4.5 ± 1.6 (33)
	**long R**	11	266.3 ± 22.3 (6.3)	370.5 ± 36.6 (9.2)	104.2 ± 26.1 (21)	309.5 ± 29.1 (7.7)	306.0 ± 33.1 (9.4)	331.2 ± 36.6 (8.6)	7.74 ± 1.4 (15)	14 ± 3.3 (24)
***sLFdown***		37	83.12 ± 24.0 (25)	202.2 ± 15.9 (5.3)	119.1 ± 22.8 (13)	180.1 ± 20.4 (11)	110.6 ± 22.1 (17)	147.7 ± 20.8 (6.5)	0.55 ± 0.1 (18)	0.0 ± 0.0 (NA)
***sLFconst***		41	341.0 ± 30.4 (2.9)	412.0 ± 32.1 (2.9)	71.01 ± 23.7 (27)	383.8 ± 32.8 (5.0)	370.8 ± 31.2 (6.0)	376.8 ± 28.5 (2.6)	0.91 ± 0.2 (16)	0.3 ± 0.7 (NA)
***sLFconst2***		36	589.8 ± 44.3 (6.3)	713.2 ± 62.2 (4.9)	123.4 ± 40.6 (17)	673.4 ± 45.4 (5.0)	623.6 ± 57.0 (6.9)	627.7 ± 47.0 (6.4)	0.76 ± 0.2 (22)	0.1 ± 0.3 (NA)
***Minter***		16	869.1 ± 210 (23)	1153 ± 103 (8.2)	284.1 ± 124 (41)	1135 ± 99.4 (7.7)	917.9 ± 210 (22)	1049 ± 121 (11)	1.58 ± 0.4 (19)	0.6 ± 0.8 (0.0)
	***MiSG3***	6	1137 ± 30.0 (1.3)	1276 ± 39.4 (1.2)	139.8 ± 43.1 (32)	1254 ± 38.0 (2.3)	1184 ± 34.5 (0.8)	1198 ± 33.0 (1.8)	1.18 ± 0.4 (28)	1.2 ± 0.9 (100)
	***Mio***	10	708.6 ± 42.5 (4.0)	1079 ± 43.2 (2.0)	370.6 ± 59.3 (10)	1064 ± 42.5 (2.0)	758.2 ± 37.9 (4.0)	958.9 ± 38.4 (2.0)	1.82 ± 0.2 (3.0)	0.3 ± 0.5 (NA)
***sup***		8	457.3 ± 13.2 (1.9)	537.3 ± 7.46 (0.5)	80.07 ± 8.55 (8.6)	481.6 ± 16.5 (2.0)	520.9 ± 6.6 (0.4)	504.8 ± 19.4 (1.4)	0.31 ± 0.1 (13)	0.0 ± 0.0 (NA)

### Semi-automated clustering

**Figure 4:**
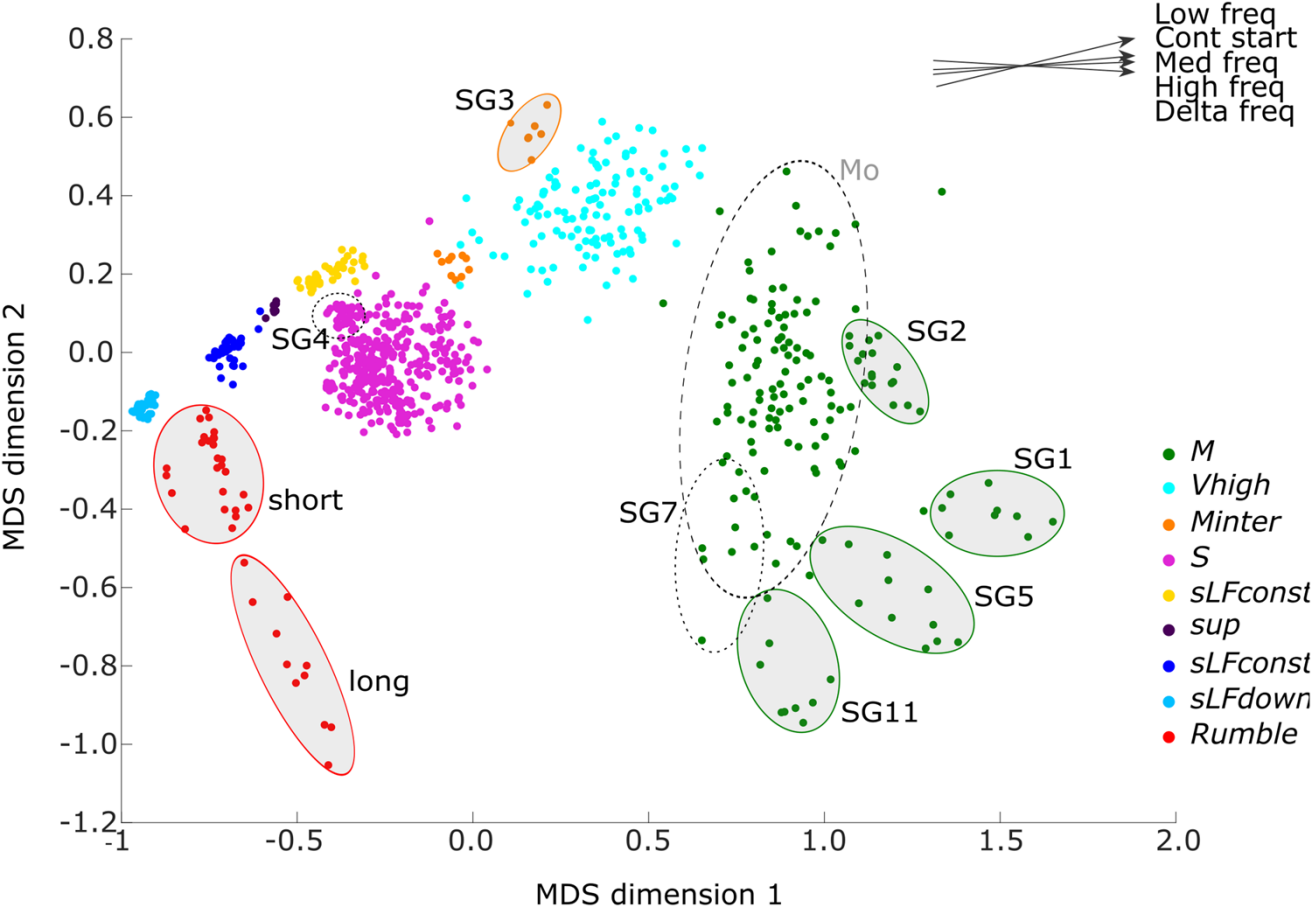
Multidimensional-scaling visualisation of 804 units from 13 song groups. Each dot represents a single unit, colour-coded according to its membership in one of the 9 unit types identified through manual analysis. Bold ellipses highlight subcluster from the MDS projection and correspond to the manual classification of song groups (except for rumble where text indicates subclusters based on duration). Dotted ellipses highlight specific unit groups and cluster mentioned in the Results. Top right corner represents the relationship between MDS axis and significant acoustic variables. The slope of the line is an indication of the correlation value and the arrow gives the direction of increasing values for the selected variables.

The MDS map globally reflected the output of the manual analysis in terms of clustering the units into separate types. Interpretation of the distances and relative positions of the clusters from the two-dimensional MDS plot was regarded as valid based on the values of R squared (R^2^ = 0.99) and goodness of fit (stress = 0.066). Close relationships (R^2^ > 0.7) were found between the first MDS axis and the variables *median frequency, high frequency*, *low frequency, contour start and frequency bandwidth*, suggesting that this dimension was strongly related to the global positioning of the unit in the frequency domain. The second MDS axis was less correlated to the acoustic variables, although correlations with *inflections* and *duration* were noticeable (R^2^ = 0.3–0.4) (Fig. 4).

Units defined by the manual procedure appeared to be well separated on the MDS map. Short units (*sLFdown*, *sLFconst*, *sup* and *sLFconst2*) were closely positioned along the first dimension, i.e. they separated along a frequency gradient, respectively from lower to higher frequency (Fig. 4). Analysis at the song group level of the *S* cluster did not reveal any clear structuring although in some SGs the units showed a tendency to aggregate in the same spatial location in the cluster (e.g. SG4, Fig. 4 and Supplementary Fig. S4a online)*. Vhigh* clusters presented a higher degree of scattering that could reflect the inaccuracy of measurements due to lower SNR of this unit type. *Rumble* were displayed along a continuum on the second dimension of the MDS map, with two subtypes based on duration parameters (short < 5 s and long > 5 s).

Unlike the densely grouped *S* units, the *M* units were more scattered. In the context of song sharing, this unit type was manually defined as a broad category, and high variability on some acoustic parameters (especially duration, inflection and frequency bandwidth) was consistently observed. The MDS projection suggested that *M* units formed five distinct subtypes (Fig. 4 and Supplementary Fig. S4b online). The map showed one large and scattered cluster (*Mo*) containing 68% of the *M* units from 9 song groups overlapping each other, and 4 smaller separated clusters, each one containing *M* units from a specific song group. *MSG11* was characterised by a very high number of inflection points and a reduced bandwidth (respectively 17 and 501 Hz, Table 2). The second cluster grouped *MSG5* units and shared similar characteristics with *MSG11* in terms of duration but displayed a three-time higher bandwidth, and only half of the inflection points. *MSG*1 had the longest duration, and intermediate values between *MSG11* and *MSG5* for inflection points. *MSG2* was grouped together based on higher values for all parameters compared to the large cluster *Mo*. Inside the 5th cluster *Mo* containing all the other SGs, fine scale structuring was visible for some song groups, as noted during the manual analysis. Interestingly, SG7 was positioned at the *Mo* cluster edges (Fig. 4 and Supplementary Fig. S4b online), in a transition position between *Mo* and the *MSG11* clusters and visual observation of the *MSG7* unit contour shapes confirmed that MSG7 shared characteristics both with the large *Mo* cluster (large bandwidth, reduced number of inflections) and *MSG11* cluster (reduced bandwidth, high number of inflections).

**Figure 5:**
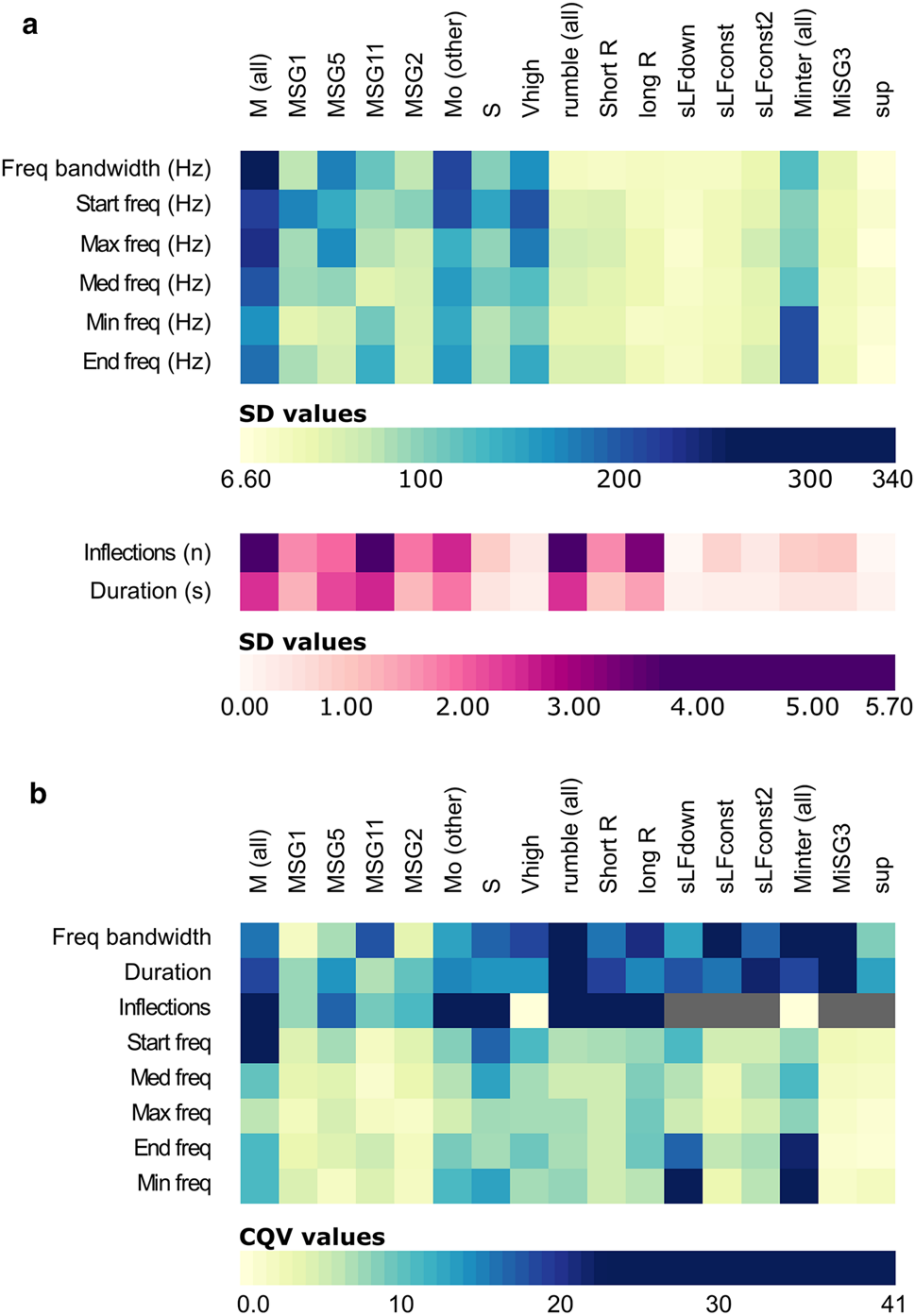
Heatmap visualisation of the dispersion of values per parameter and unit type. (**a**) Standard deviation (SD) (Inflection and Duration plotted apart due to smaller range of values). (**b**) Coefficient of quartile variation (CQV) (Grey colour indicates that the cqv values could not be computed). Note that the SD has the same unit as the parameter it is computed from and the CQV has no unit.

Standard deviation (SD) values for the *M* unit acoustic parameters were higher than for the other unit types, and still superior to *S* type values when considering only the *Mo* cluster (Table 2 and Fig. 5a). Short units had low SD values*.* Globally, the coefficient of quartile variation values per parameter showed that inflection, duration and frequency bandwidth were the most dispersed around the mean (Table 2 and Fig. 5b).

Clusters from the MDS map were used in combination with the manual analysis to create an improved classification of unit types and its validity was assessed using the silhouette coefficient. Values of 0.49 for both the clustering of *M* units and the Except-*M* units were higher than the values obtained for the hierarchical clustering for the same number of clusters (respectively 0.40 and 0.28) (Supplementary Fig. S5 online). A higher silhouette value represents a better clustering solution where each object (unit) is closer to its own cluster centroid. Silhouette values from the MDS map clusters were also higher over a range of cluster numbers, with one exception that clearly oversimplified the grouping of the units into only 3 or 4 distinct clusters.

### Biphonation patterns on M and S units

All the analysed song groups presented a biphonation on one or two unit types. Biphonation presented strong similarities between some song groups, and could be divided into 3 and 4 major types for *M* and *S* units respectively (Supplementary Table S6 online). Close ups of the units are shown in Supplementary Fig. S7 online. The main recurrent biphonation type on *M* and *S* units was a tonal frequency modulated downsweep, respectively present in 6 and 7 song groups. *M*bi1 overlapped in time with the whole *M* unit, and sometimes even with the following *S* unit. *S*bi1 tonal downsweep sometimes included frequency modulation. Remarkably different, consisting in short broadband “whoops” in sequence, *M*bi2 presented a highly consistent and repetitive pattern on SG11 and SG13 but variable temporal characteristics in other song groups (SG2, SG8 and SG10). A similar biphonation pattern was present on *S* unit from SG1 (*S*bi2). *M* from SG4 showed a distinct type of biphonation, a short constant tonal around 130 Hz with 3 harmonics, present at the end of the unit. Altogether, biphonation patterns resulting from the combination of biphonation types on *M* and *S* units were unique for almost half of the song groups.

## Discussion

Bowhead whales singing behaviour has been studied for more than 3 decades and an increasing diversity in their song repertoire has been described from the simple patterned moan sequences reported by Ljungblad et al.^34^ to the remarkably high song diversity described for the Spitzbergen population^22^. Although previous studies provided song or unit descriptions, there has been little discussion about variability and consistency in bowhead whale singing display and its impact on our understanding of bowhead whale acoustic ecology in a mating context.

Bowhead whale songs were almost continuously present from November 2013 to March 2014, with a peak of vocal activity between December 2013 to February 2014. Previous studies in the Fram Strait^21,22^, also described intense singing activity of bowhead whales in winter months, suggesting that this location was a mating ground for the Spitsbergen population^33^. Our data, recorded approximately 170 km from the Fram Strait, provides compelling acoustic evidence that the spatial range of the mating ground of this critically endangered population would likely extend from the western Fram Strait to the Northeast Greenland coastal area, hundreds of kilometres more west than previously reported.

Past studies describing the bowhead whale repertoire from different populations have reported the occurrence of the same song overlapping in time, demonstrating that bowhead whales do share songs^2,3,8^. Elaborating on this finding, Johnson et al.^8^ estimated that during the migration of whales from the Bering-Chukchi-Beaufort population, a song type could be shared by up to nine animals. Temporal overlapping of songs in our data revealed that at least three animals were sharing the same song (Fig. 2). Structural characteristics of these shared songs were used to identify song groups based on similar unit types arranged in comparable temporal pattern, leading to the selection of 13 song groups. Analysis of the song groups revealed variability at multiple hierarchical levels of the songs, from units to phrases.

Variations in a given unit type have been described in previous acoustic studies of bowhead whale songs. Evolution of unit structure (loss of part of the unit, splitting into smaller units) or frequency (e.g. shifting) has been reported from all the populations that have been acoustically studied so far^2,3,8^. Our analysis revealed two major paths for intra-unit type variability, that were measured in terms of standard deviation and coefficient of quartile variation and were readily noticeable on the MDS maps. The first path of variation was related to duration and inflection points and was especially noticeable on two unit types, *M* and *rumble.* These two unit types both had a distinctive frequency contour displaying multiple inflections, thus variation in duration was coupled with variations in the number of inflection points. The second path was linked to variability in frequency parameters with variations mainly related to frequency bandwidth. Gradual changes on one or both of these paths of variation eventually led to the appearance of new unit types, and therefore larger vocal repertoire. Novel or complex vocal repertoires have been identified as important traits influencing female sexual selection in several studies on birds (e.g.^35,36^) and in a similar way, sexual selection might be one of the mechanisms underlying innovation in humpback whale song^37,38^.

**Figure 6:**
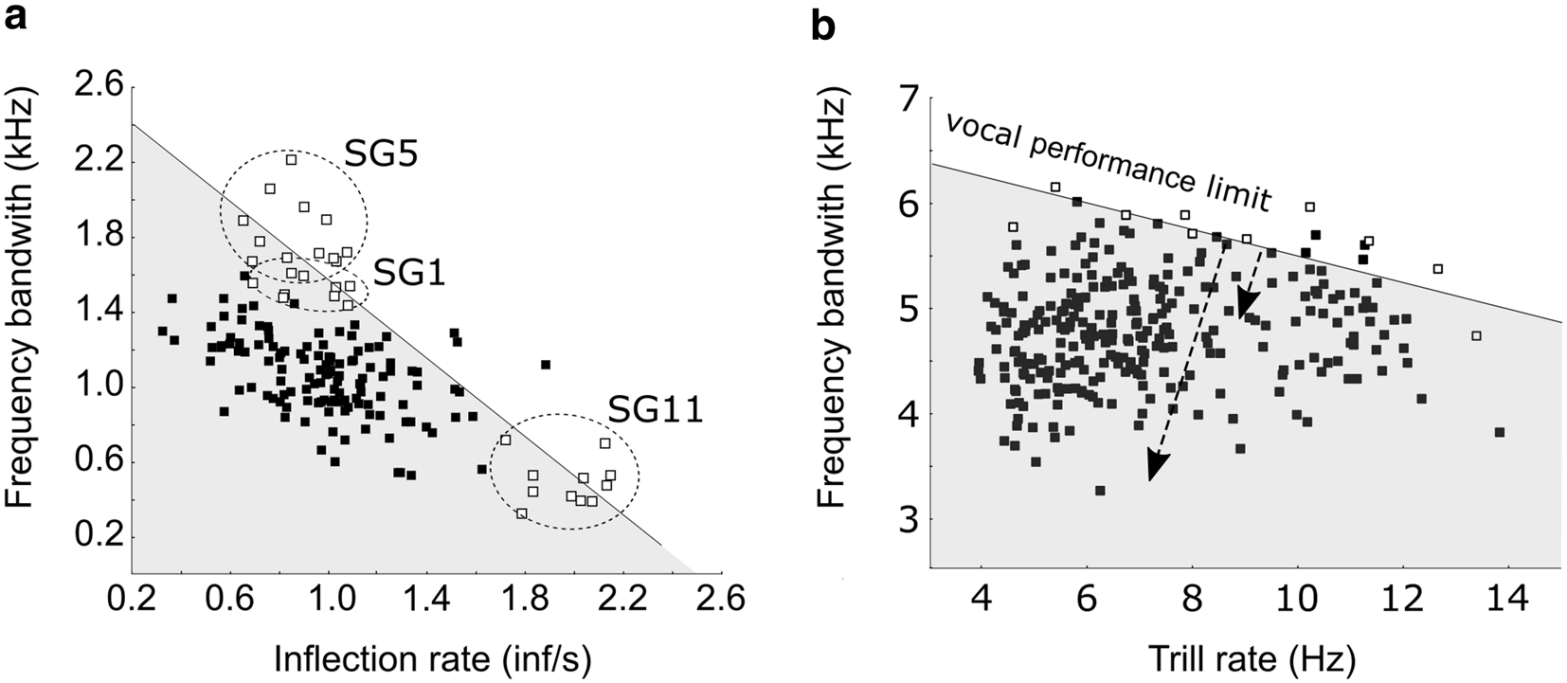
Vocal performance limit approach in 2 species, the bowhead whale and the swamp sparrow (*Melospiza georgiana*)*.* (**a**) Inflection rate by frequency bandwidth for 158 M units recorded from bowhead whales (this study). Ellipses highlights song groups mentioned in the text. (**b**) Trill rate by frequency bandwidth for 280 songs recorded from 91 male swamp sparrows^40^. Open circles represent the points that were used to calculate the upper-bound regression line. The upper-bound regression line represents the vocal performance limit. Low deviation from this line indicates a high-performance song. High deviation indicates a low-performance song (arrows). Grey areas represent the “acoustic space” that vocalisations occupy^41^.

While ecological and behavioural functions of songs in bowhead whales are still unclear, the presence of intensive singing during the reproductive season indicate a function in sexual advertising. This would correspond to a polygynous mating strategy as seen in humpback whales (where one male mates with multiple females), although the exact nature of the bowhead whale mating system is still unclear to researchers^17^. In this context, a possible advantage of displaying calls with longer duration could be linked to an increase in stamina with age or as an honest advertisement of physical fitness as it has been described in bearded seals, *Erignathus barbatus*^39^. Variability in inflection points could similarly be associated to fitness since highly modulated sounds require higher motor control of the vocal apparatus. Interestingly, for most of the unit types, variability was contained inside a unique cluster corresponding to a single unit type (i.e. unit types were separable in the feature space as they had little to no overlap). However, combined variability on both frequency and modulation/duration parameters led to a gradual split of *M* units into subtypes, some of them matching the song groups labels. The *M* unit type, possessing characteristic of long duration, numerous inflections points and variable bandwidth could be identified as the unit that best represents abilities of the animal to produce physically challenging sounds. This would be similar to trilled songs in songbirds where production of trills, expressing vocal performance, defines a constraint on the song, represented by a trade-off between trill rate and frequency bandwidth^40–42^ (Fig. 6). The upper boundary of this trade-off represents a vocal performance limit and songs containing trills with acoustic characteristics close to this limit are considered to express high vocal performance^40–42^. In the context of sexual selection, vocal performance has been related to male quality assessment by female birds and to male-male competition^40,43^. It appears that highly modulated sound produced by bowhead, such as the *M* units studied here, face a similar trade-off between number of inflection points (analogue to trill repetition rates) and the frequency range they can span (Fig. 6). Song groups containing *M* units at the limits of the performance constraints, such as SG1, SG5 and SG11, could then be associated with higher vocal performance (Fig. 6). If songs are used in a mate choice context, the presence of units with numerous inflections could function as an honest indicator of mate quality, by virtue of the “inflection rate/frequency band” trade-off. As such, these units could carry a higher level of inter-individual variability expressing different levels of fitness, and additionally higher levels of intra-individual variability in relation to the difficulties of accurately producing and reproducing such sounds.

An additional source of unit-level variability described here comes from the presence of biphonated signals. Recent acoustic studies confirmed that bowhead whales use biphonation^4^ and that the use of biphonated signals can be quite common in bowhead whale songs^8^. All the song groups studied here showed biphonation on one or two unit types, with the occurrence of different biphonation types including continuous whistle-like sounds, short impulsive broadband sounds and harmonics signals. Functions of biphonated signals remain unclear and they have been suggested to encode information on size^44^, individuality^45–47^ or localisation of the signal emitter^48,49^. Mastering the production of biphonated signals, which require high vocal control of the laryngeal apparatus (*Musculus diverticuli laryngei*)^50^, could function as another honest signal of mate quality in a context of reproductive display^4^. If one structure is the source of two simultaneous sounds, then producing two very different sounds i.e. decoupling mechanisms of air movements in the larynx, would require strong vocal skills. Broadband discrete impulsive signals coupled with continuous *M* units could be more difficult to produce than whistle-like types. As such, the presence of type *M*bi2 could indicate older or more experienced singers and, interestingly, this biphonation type occurred concurrently with *M* units than can be associated with higher vocal performance (SG11). Similarly, recent studies on bowhead whales’ closest relatives, the North Atlantic right whales, have linked age with increasing structure in non-linear phenomena, from disorder (deterministic chaos) to increased control (biphonation and subharmonics)^51^.

On the upper level of the song hierarchy (phrases), temporal arrangement of units in phrases showed regular patterns of association between units in stable phrases. However, while phrases containing *M* and *S* were present in every song group, phrases containing the short alternating units (*sLFdown, sLFconst, sLFconst2, sup*) were only present in half the song groups. The duty cycle used in this study (2 min on, 38 min off) could have resulted in recording only partially the variability of each song group. The average song duration of a bowhead whale song is considered to be approximately one minute^1,3,8,21^ but songs lasting almost two minutes have been reported. Therefore, in our study, most of the time, only one entire song was present in the file, and variability at the song bout level (differences in successive iterations of the same song by the same animal) could not be assessed. Nevertheless, the aforementioned phrases containing the short alternating units were present in half of the studied song groups, and can be considered as common and part of the song structure. In humpback whales, the only other cetacean species to produce such complex songs, omission or insertion of entire themes is considered part of the natural evolution of song types and themes present in 50% or more of the songs were included in song sequence study^14^. Similarly, temporal evolution of a song type over a period of a few months has been previously described in bowhead whales. Delarue et al.^3^ reported the observations of primitive versions occurring during the initial development of the song until the final, stable version emerged. The overall song variability suggested a low level of constraint in the song structure (e.g. units missing, variability in the repetition rate of units, presence or absence of a phrase). In humpback whale songs, phrase structure is reported to be much more flexible than in bird songs and can even be progressively modified within individual songs^14,52^. While there are inherent recording limitations when conducting acoustic studies in the high Arctic, longer duty cycles are required to quantitatively describe song structure variability and evolution over a season.

To describe and quantify variability at unit level and to allow the description of song structure, we have used a semi-automated method combining multidimensional scaling maps and human expertise. MDS maps showed unit clusters corresponding well to human classification into nine distinct unit types. Based on this agreement, MDS was used to investigate subclustering in the *M* unit type. Units were positioned along a continuum on the MDS axes, following a gradation in the frequency, modulation and duration parameters of this unit type, which complicated the classification into subtypes. Graded vocal repertoire has been described for many mammalian taxa such as primates^53^, elephants^54^, sirenians^55^, odontocetes^56–58^, and mysticetes including humpback^59^ and right whales^60,61^. Graded repertoires are challenging for classification tasks since there is no clear boundary where a grouping begins or ends. Manual (human) classification of animal vocalisations usually leads to accurate results^62^, but with a strong subjective component, whereas automatic methods can add objectivity and standardisation. It appears that a combined method such as the one we described here can greatly help the classification process at the unit level. The next step forward towards standardisation of bowhead whale song studies would be applying automated methods that have been validated on other species. For example, the Levenshtein distance, a similarity analysis that compares vocal sequences, has been successfully applied to humpback whale songs to objectively quantify differences among groups of songs^63–65^.

Earlier studies suggested that bowhead whales, unlike all other singing whale species, entirely change their song repertoire from year to year. This feature has been reported for the Bering-Chukchi*-*Beaufort (BCB) population on their migration route^1,3^ and for the Eastern Canada-Western Greenland (EC-WG) population in Disko Bay in spring^5^. It is likely that these studies only sampled a small part of the song repertoire, neither locations being wintering grounds where most of the singing is thought to occur in relation to reproduction^66^. A recent study from the Spitsbergen population analysed data from a probable mating area in the Fram Strait during four successive winter seasons. As predicted, researchers recorded and described a much higher diversity of songs. Again, the results highlighted the lack of recurrence of song types from year to year, indicating that bowhead whales completely renew their singing repertoire each year^22^.

**Figure 7:**
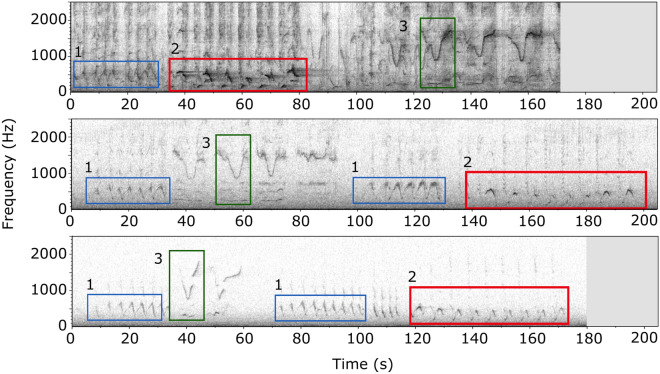
Examples of 3 bowhead whale songs recorded from the Greenland Sea. Top, song recorded in Northeast Greenland on the 11 of November 2013 (this study). Middle and bottom, songs recorded in the Fram Strait, respectively from November 2013 and December 2012^69^ (Spectrogram parameters: 1024 pts FFT, 50% overlap, Hann window). The numbers correspond to a single unit or a sequence of units: 1 is a long frequency-modulated sound around 500 Hz with a distinctive growling on the highest parts of the signal; 2 is a sequence of alternating downsweep and upsweep units; 3 is a “V” shaped unit with a flat biphonation.

**Figure 8:**
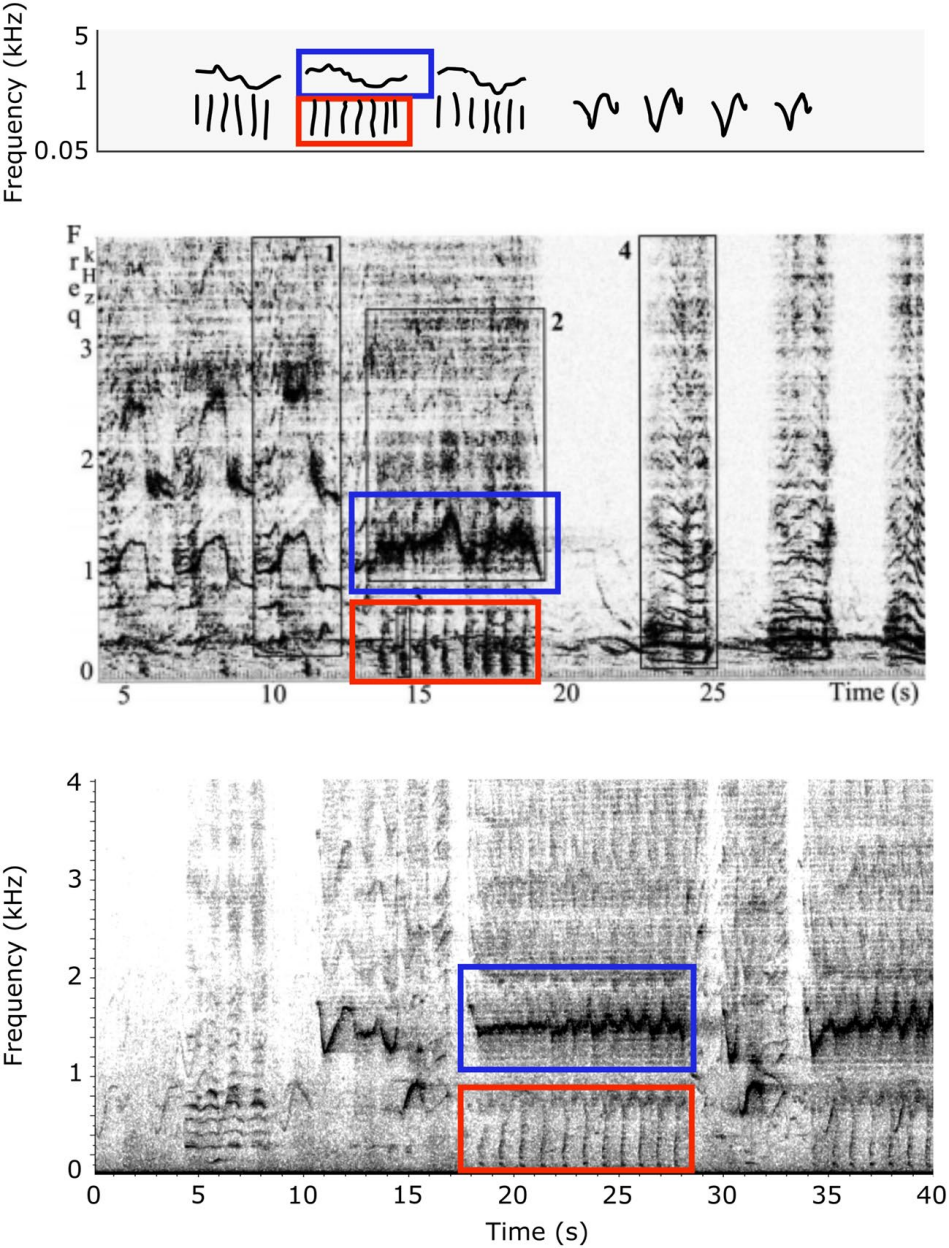
Examples of 3 bowhead whale songs displaying a highly similar biphonated unit. Top, song from the Chukchi Sea recorded in 1988^1^. Middle, song from the Chukchi sea recorded in December 2007 (^3^ adapted from, reproduced with the permission of the authors and the Acoustical Society of America). Bottom, song from the Greenland Sea recorded in December 2013 (this study). (Spectrogram parameters: 2048 pts FFT, 50% overlap, Hann window). The blue box corresponds to the long whistle-like vocalisation, with multiple inflections (*MSG11* described in this study). The red box shows the associated biphonation, a rapid sequence of shorts grunts or “whoops”.

Nevertheless, our results suggest that the high variability reported in this study could hinder the recognition of units and phrase types that are potentially reused from year to year, in a dynamic and progressively evolving singing display. Although songs may be considered different between years (depending of the level of variability researchers select to assess song diversity), some unit types and patterns of association between unit types may be maintained over time. Acknowledging this inherent variability could help to track patterns in units and song presence at different temporal and geographical scales. For example, close examination of songs from the Spitzbergen population from our dataset and from the Stafford et al.^22^ study revealed that identical units forming highly similar songs were present at the two locations (170 km apart), during the same time period (December 2013–January 2014) and moreover, were present at the same location in the Fram Strait during two consecutive years (Fig. 7). Furthermore, we discovered the presence of highly similar units in our dataset from Northeast Greenland and in published song studies from the BCB population. The *MSG11* unit described here is identical to the warble unit characterised in Delarue et al.^3^ and to the high-frequency whistle reported in Würsig and Clark^1^. Figure 8 shows that units from theses 3 studies share similar median frequency (around 1500 kHz), shape (multiple inflections), and an identical highly specific biphonation pattern (broadband grunt or “whoop”). Additionally, the 1988 song described by Würsig and Clark^1^ is composed by the aforementioned high-frequency whistle associated with another unit that closely resembles the *S* unit described here, both in terms of frequency parameters and contour shape (Fig. 8).

These findings are highly relevant in the context of population exchanges questions that arose from recent studies of the Spitzbergen population^22,23^. Whether the individuals recorded from the Greenland Sea are remnant of the nearly extirpated Spitzbergen population or immigrants from other populations (BCB or EC-WG) is not known. Drastic sea ice loss in the high Arctic has already made contact possible between once ice-separated bowhead populations and the continuous increase of this phenomena is likely to open new areas for bowhead whale migration^67^. Tracking acoustic similarities at the unit or phrase level will help to gather acoustic evidence revealing these movements.

Bowhead whales are undoubtedly amongst the most prolific singers, and the high diversity of the singing repertoire has just begun to unveil. In order to build up our understanding of bowhead whale acoustic ecology, we propose that future acoustic studies should consider (i) further developing automated methods using objective categorisation to facilitate inter study comparisons, (ii) shifting the framework of acoustic studies from a song-based to a unit or phrase-based approach as it has been suggested for humpback whales^19^ to investigate song similarities in the context of song sharing, song evolution and cultural transmission, (iii) conducting collaborative studies to track acoustic patterns at larger temporal and geographic scales.

## Supplementary Information

Supplementary Information.

## Data Availability

The datasets generated during and/or analysed during the current study are available in the LIDO repository, http://www.arctic.listentothedeep.com/.
