# Supplementary material for: Contribution to unravel variability in bowhead whale songs and better understand its ecological significance
Source: Sci Rep. 2021 Jan 8;11:168. doi: 10.1038/s41598-020-80220-5 (PMC7794550; doi:10.1038/s41598-020-80220-5)

Contribution to unravel variability in bowhead whale songs and better understand its ecological significance

Erbs, F.^1^, van der Schaar, M. ^1^, Weissenberger, J. ^2^, Zaugg, S.^1^, André, M. ^1*^

Supplementary Figure S1. Weekly occurrence of bowhead whale song detections. 11 files per 24 hours were manually analysed for song occurrence. The maximum value for weekly detection (maximum weekly count) is 77 (11 files x 7 days).


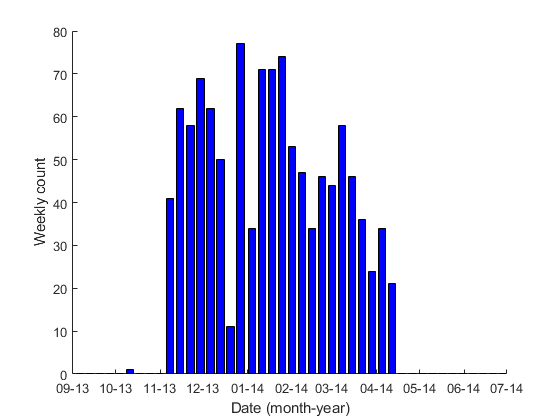


Supplementary Figure S2. Temporal occurrence of the 13 song groups analysed.


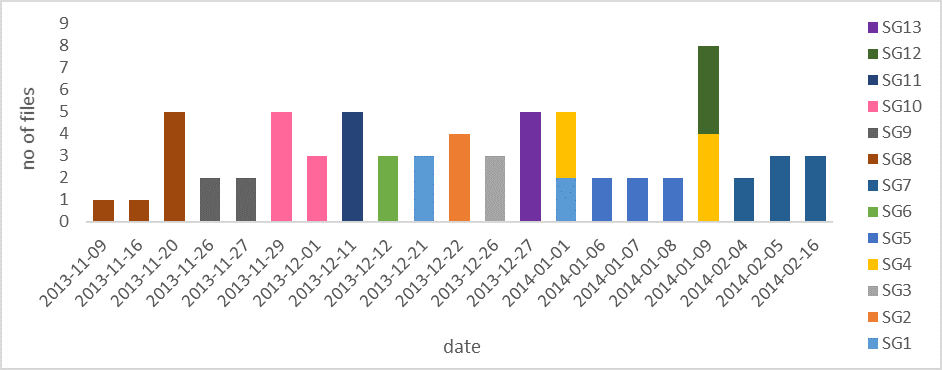


Supplementary Figure S3. Contour sketch examples of the 9 unit types, based on their fundamental frequency. Each slice represents 10 seconds on the time axis.


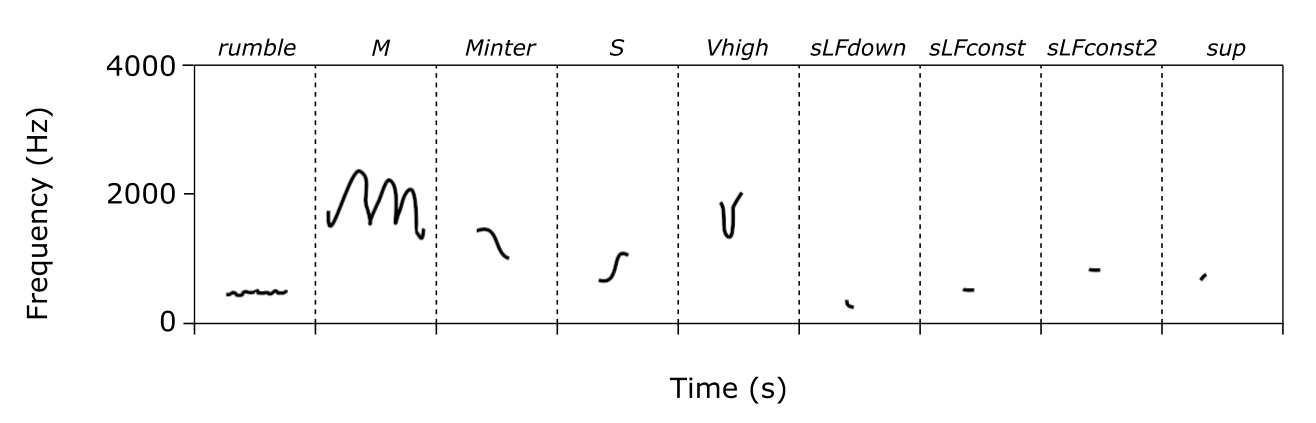


Supplementary Figure S4. Multidimensional-scaling visualization for unit types *M* and *S*. a) MDS of 351 *S* units from 13 song groups. b) MDS of 158 *M* units from 13 song groups. Each dot represents a single unit, shape-coded according to its song group membership in one of the 9 unit types identified. Grey ellipses highlight song groups boundaries. Transparent ellipse indicates cluster *Mo* in b).


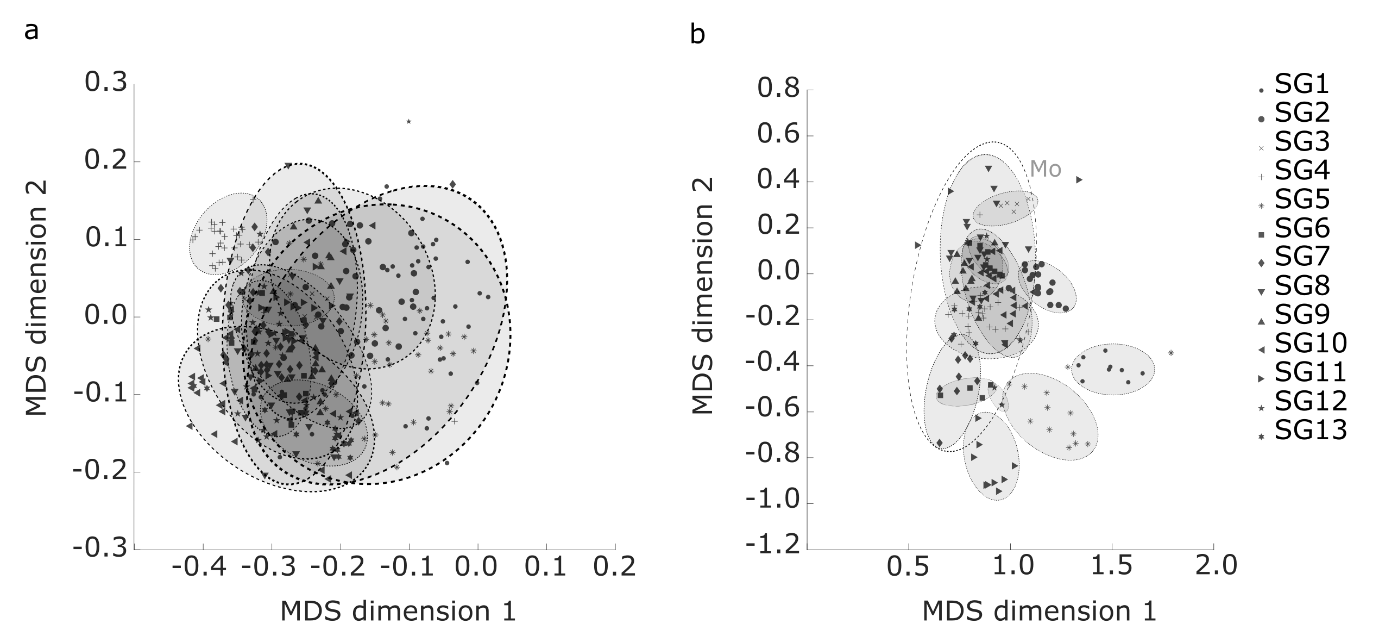


Supplementary Figure S5. Silhouette values corresponding to the MDS semi-automated clustering (crosses) and to the hierarchical clustering (solid lines) results. Colours correspond to data subsets.

*
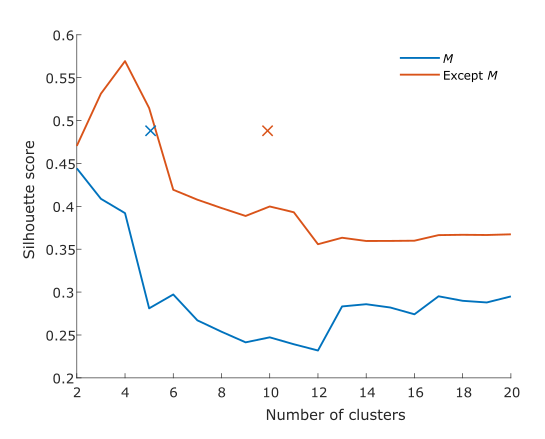
*

Supplementary Table S6. Description of biphonation signals on units *M* and *S* and occurrence in song groups.

| Biphonation type | Description | Frequency range (Hz) | Song group |
| --- | --- | --- | --- |
| Mbi1 | long tonal frequency modulated downsweep | 100 - 800 | SG3, SG5, SG6, SG7, SG9, SG12 |
| Mbi2 | short (< 300 ms) “whoops” in sequence | 100 - 900 | SG2, SG8, SG10, SG11, SG13 |
| Mbi3 | short constant tonal with 3 harmonics | 120 - 130 | SG4 |
| Sbi1 | tonal downsweep that sometimes included slope modulation | 100 - 400 | SG1, SG5, SG6, SG7, SG11, SG12, SG13 |
| Sbi2 | biphonation similar to the Mbi2 “whoops” | 100 - 300 | SG1 |
| Sbi3 | tonal moan with harmonics or subharmonics | 100 - 300 | SG2, SG8, SG10 |
| Sbi4 | short tonal upsweep before *S*bi1 | 300 - 350 | SG2 |

Supplementary Figure S7. Spectrogram screenshot of unit types M and S, belonging to each of the 13 song groups analysed. Top, unit types M; Bottom, unit type S. Note the biphonation patterns present on most of the units.


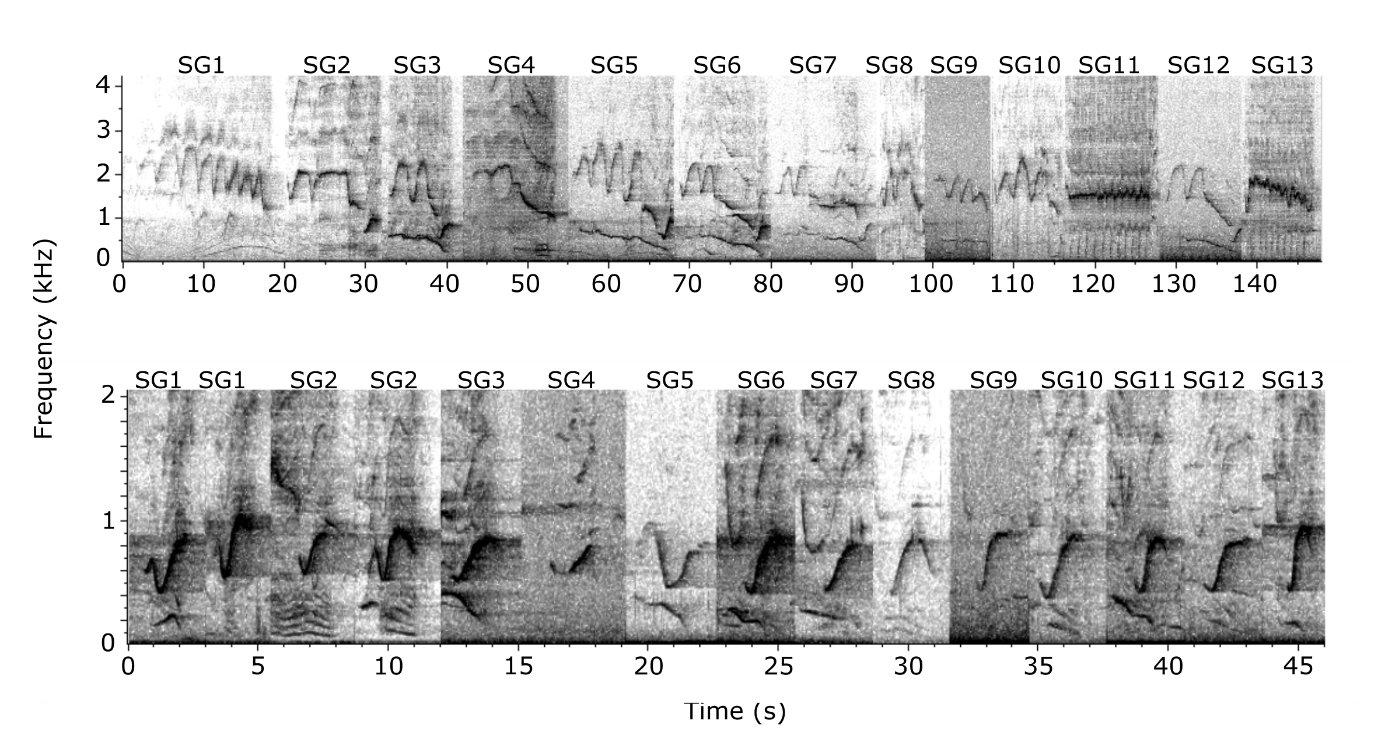

Supplement: Supplementary file 1 — Supplementary Information. [file 41598_2020_80220_MOESM1_ESM.docx]
